# Kinetic Studies and Mechanism of Hydrogen Peroxide Catalytic Decomposition by Cu(II) Complexes with Polyelectrolytes Derived from L-Alanine and Glycylglycine

**DOI:** 10.1155/2010/643120

**Published:** 2010-07-18

**Authors:** Spyridon Skounas, Constantinos Methenitis, George Pneumatikakis, Michel Morcellet

**Affiliations:** ^1^Inorganic Chemistry Laboratory, Department of Chemistry, University of Athens, Panepistimiopolis, 15771 Athens, Greece; ^2^Laboratoire de Chimie Macromoléculaire, UMR 8009 CNRS, Université des Sciences et Technologies de Lille, 59655 Villeneuve d'Ascq, France

## Abstract

The catalytic decomposition of hydrogen peroxide by Cu(II) complexes with polymers bearing L-alanine (PAla) and glycylglycine (PGlygly) in their side chain was studied in alkaline aqueous media. The reactions were of pseudo-first order with respect to [H_2_O_2_] and [L-Cu(II)] (L stands for PAla or PGlygly) and the reaction rate was increased with pH increase. The energies of activation for the reactions were determined at pH 8.8, in a temperature range of 293–308 K. A suitable mechanism is proposed to account for the kinetic data, which involves the Cu(II)/Cu(I) redox pair, as has been demonstrated by ESR spectroscopy. The trend in catalytic efficiency is in the order PGlygly>PAla, due to differences in modes of complexation and in the conformation of the macromolecular ligands.

## 1. Introduction

The formation of complexes between macromolecular ligands and transition metal ions has been widely investigated [1–6]. Synthetic macromolecular systems, offer the possibility of modelling the complexation and reactivity of metal ions with biological ligands [7]. The systems especially that are made up of synthetic polyelectrolytes could be considered as simple but representative models for studying biological ligand-metal interactions [7–9]. The thermodynamic and structural characterization of these metal ions complexes would help in understanding the mechanism and the binding mode of the metals both to proteins and to substrate molecules.

The decomposition of hydrogen peroxide has been used as a model reaction for the investigation of the catalytic activity of various metal complexes and has also been studied as a catalase model, although the catalytic mechanism has not been thoroughly elucidated [1, 2, 10–21]. It has been known for about a century that the decomposition of H_2_O_2_ to H_2_O and O_2_ is drastically accelerated by many metal ions [14–19]. Complexes of copper (II) especially with various ligands acting as catalysts have been investigated in depth and disagreements over mechanistic details, involving intermediate radicals or complexes, have lasted for decades. The formation of copper peroxide complexes both in acidic and alkaline solutions has been confirmed and a mechanism not involving any radicals has been suggested [17, 18]. On the other hand, the existence of OH^.^ radicals in the decomposition of H_2_O_2_, catalyzed by Cu(II), in alkaline media in the presence of biological reductants has been, convincingly, demonstrated [20–22]. The difference in reactivity of Cu(II) complexes towards H_2_O_2_ is due to the change in the redox potential of Cu(II) ions as a result of ligation with different ligands [23]. It was supposed that a superoxide-copper (I) complex is formed [HO_2_-Cu(I)(Ligand)] from the complex [HO_2_-Cu(II)(Ligand)] in which an instantaneous electron transfer occurs [21]. Other investigators suggested the formation of a Cu(III)-peroxo complex, as a consequence of the interaction of Cu(II) ions with H_2_O_2_, this species producing highly reactive OH^.^ radicals [24–30]. Recently, a mechanism involving both Cu(I)/Cu(II) and Cu(II)/Cu(III) redox pairs has been reported [31]. It has also been proposed that in slight alkaline and neutral pH region, the decomposition of H_2_O_2_ by copper complexes may proceed by a combination of a molecular mechanism, in which a presumed intermediate species [CuL(HO_2_^−^)^+^], reacts with [HO_2_^−^], a free-radical mechanism involving reversible oxidation reduction of a cupric-cuprous couple, and the formation of free radicals, for example, HO_2_^.^ and HO^.^, leading to a chain reaction [32]. Other researchers have mentioned the formation of a peroxo-copper complex (brown compound) in the homogeneous and heterogeneous H_2_O_2_ decomposition with different Cu(II) complex ions that are by themselves active catalysts for H_2_O_2_ decomposition [33].

**Scheme 1:**
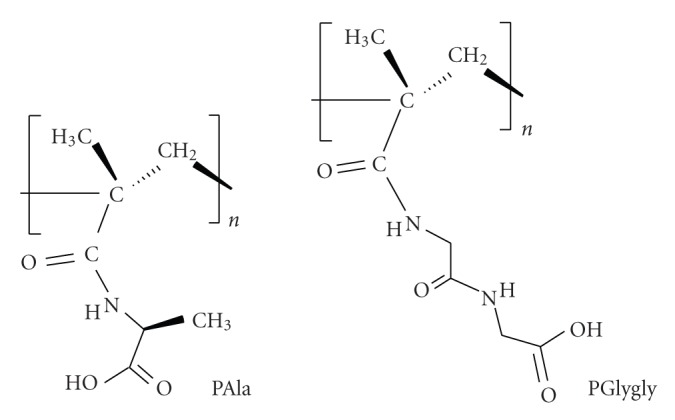
The polyelectrolytes poly-N-methacryloyl-L-alanine (PAla) and poly-N-methacryloyl-glycylglycine (PGlygly).

A series of polymers that are referred to as “nonpeptide amino acid-based polymers” or as “amino acid-derived polymers with modified backbones” [34] was previously synthesized and shown to exhibit polyelectrolyte and metal complexation behavior [35–40]. In previous works it has been shown that the complexes of Cu(II) with polyelectrolytes derived from glutamic and aspartic acids exhibit substantial catalytic activity in the decomposition of H_2_O_2_ in alkaline media [41, 42]. Key feature in these systems is the coordination of the ligands to Cu(II) through two amidic N^−^ from adjacent amino acids [41, 42]. In the present work, we investigate the catalytic activity of Cu(II) complexes with polyelectrolytes derived from alanine, PAla, and diglycine, PGlygly, (Scheme 1) towards the H_2_O_2_ decomposition in alkaline solutions. A suitable mechanism is proposed to account for the kinetic and ESR spectroscopic studies.

## 2. Experimental Section

### 2.1. Materials and Methods

All chemicals were of analytical reagent grade and were employed without further purification. The poly(N-methacryloyl-L-alanine) (PAla) and poly(N-methacryloyl-diglycine) (PGlygly) were prepared by polymerization of the corresponding monomers, carried out in dioxane (p.a Merck) at 60°C with AIBN (azobisisobutyronitrile) as initiator. The monomers N-methacryloyl-L-alanine and N-methacryloyl-diglycine were prepared from methacryloyl chloride (tech. 90%, with 150 ppm phenothiazine, Aldrich Chem.Co) and L-alanine (optical purity >99, 5% NT, Fluca) and glycinoglycine (>99, 5% NT, Fluka), respectively, according to the method of Kulkarni and Morawetz [43]. Full experimental details of the synthesis of these compounds have been published earlier [35, 37]. Cu(ClO_4_)_2_·6H_2_O (Fluca) was used as the metal ion source. Water was obtained from a Milli-Q purification system (Millipore), which was feeded with doubly distilled water. The concentration of Cu(II) solutions was determined by complexometric titrations with EDTA. Working solutions of hydrogen peroxide were prepared weekly by volumetric dilution of 30% (v/v) H_2_O_2_ (AR. grade, Merck) and were standardized daily by titration with potassium permanganate. It was found that the natural decomposition rate of aqueous H_2_O_2_ solution was less than 1% in 24 h. 

The decomposition of H_2_O_2_ catalyzed by Cu(II) complexes can be kinetically monitored by removing aliquots of the reaction mixture at predetermined intervals and titrating the undecomposed H_2_O_2_ with standard KMnO_4_ solutions (0.04-0.05 N), standardized with (COONa)_2_ (primary standard). The decomposition of hydrogen peroxide was carried out in a thermostated cell at four different temperatures between 293 and 308 K (±0.1 K). The pH of the solutions was adjusted between 7 and 11 with buffer solution of H_3_BO_3_-NaClO_4_ and the appropriate quantities of NaOH 0.1 M and between 6-7 with buffer solution of phosphate. The pH measurements were performed using a digital Xenon pHmeter and a RUSSEL CMAWL/3.7/180 combined electrode. Standardization was done at 25°C with Russel buffers (potassium hydrogen phthalate at pH 4, potassium dihydrogen orthophosphate-disodium hydrogen orthophosphate at pH 7 and sodium hydrogen carbonate/sodium carbonate at pH 10). The chosen concentration range of H_2_O_2_ was 3.3 × 10^−3^ to 1.3 × 10^−2^ M. The analytical concentration of Cu(II) ranged from 1.3 × 10^−4^ to 7.8 × 10^−4^ M and the [ligand]/[metal] ratio (*R*) was chosen to be 4 and 8. The decomposition of H_2_O_2_ was halted with the addition of H_2_SO_4_. Acidification effectively halted the alkaline decomposition at the desired time and titrations with potassium permanganate in acidic media could be carried out even 1 h later without significant discrepancy.

### 2.2. EPR Measurements

The measurements were made in frozen (77 K) aliquots of the solutions of the reactions between the systems L-Cu(II) (L stands for PAla and PGlygly) (*R* = 4, [Cu(II)] = 1.0 × 10^−3^) and H_2_O_2_ at pH between 8.0 and 9.0 at 298 K, taken out at different times and carried out on a Bruker ESP-300 spectrometer (X-band) with 100 kHz field modulation, 9.3 GHz microwave frequency and equipped with a standard low-temperature apparatus. The hyperfine coupling constants and g-factors were calibrated by comparison with DPPH (2,2-diphenyl-1-picrylhydrazyl) (*g* = 2.0028).

## 3. Results and Discussion

### 3.1. Solution Structure of the Complexes

The presence of both the carboxyl and the amidic groups in the ligands (Scheme 1) gives the possibility of different complex type formation. Although the exact geometry of the formed complexes cannot be reached because of the complexity of the macromolecular structures, the atoms of the ligands which interact with the Cu(II) ions and the stoichiometry of the formed complexes can be determined. Based on the potentiometric and spectroscopic results, molecular structures for the dominant copper(II)-PAla species existing in aqueous solutions were previously proposed in [35, 36]. At pH < 4 the complex formation involves two carboxylates and water molecules (type (I) complex); at pH > 4 another complex, a chelate with the participation of a deprotonated N atom of the amide function, starts to form (type (II) complex), that gradually becomes the main species. At pH > 9 complexes which involve two deprotonated amide N are also detected (type (III) complexes). For PGlygly-Cu(II) systems, at pH < 9.5 different complexes involving carboxylates, water molecules and/or hydroxyls are detected (type (I) complexes) [39]. At pH > 9.5 complexes with the participation of deprotonated N atoms of the amide functions are, as previously, also formed (type (III) complexes, unpublished results). It must be mentioned that at pH > 9 hydrolysis, in some extent, of the above copper complexes occurs and the formed copper hydroxides do not precipitate but could be firmly bounded to the soluble polyelectrolytes [44, 45].

### 3.2. Catalytic Properties

A number of experiments were performed to study the effect of pH, H_2_O_2_, and Cu(II) concentrations and temperature on the initial rate of the catalytic decomposition of H_2_O_2_ from the PAla-Cu(II) and PGlygly-Cu(II) systems. The reaction velocities at *t* → 0 (*v*_0_) were determined graphically from the tangential slopes of the curves, at zero time, which represent the concentration of hydrogen peroxide in respect to the elapsed reaction time. Thus the initial rate, *v*_0_, was expressed as *v*_0_ = (*d* *c*/*d* *t*)_*t*→0_.

Characteristic changes in the colour of the reaction solutions take place during the course of the reactions. The clear sky blue solutions of Cu(II) complexes turn into blurred blue-green after the addition of H_2_O_2_. Gradually, they become yellow-green and when concentrations of hydrogen peroxide higher than 1.3 × 10^−2^ M are used, a yellow-brown solid precipitates from the solutions with pH > 9.5. No attempt was made to investigate the nature of this precipitate. Small bubbles of oxygen are formed during the reaction. Within a week the reaction solutions did not get the initial colour. Blank experiments were performed and no catalytic activity was observed when solutions containing only the polymers in the absence of Cu(II) were used, in the whole pH range. At the pH range under investigation copper hydroxide is precipitated in the absence of the polyelectrolytes. 

**Figure 1:**
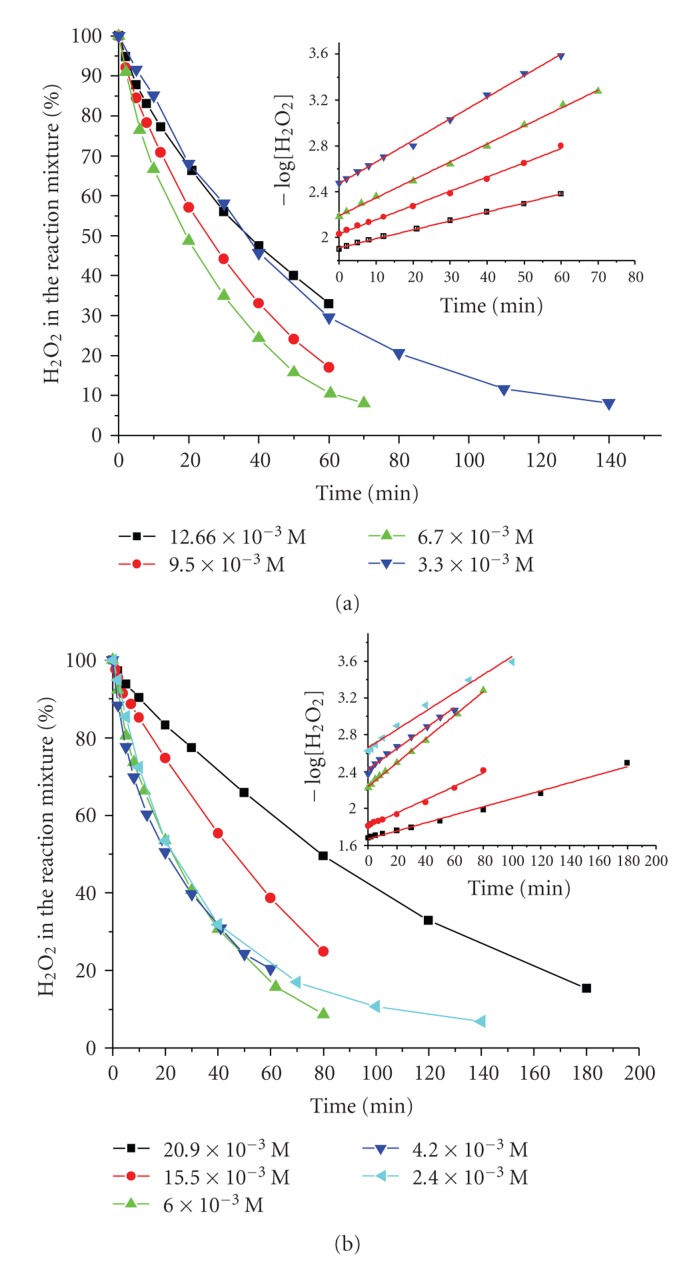
The variation of [H_2_O_2_] in the reaction mixture (% of the initial concentration) and (insert) negative logarithm of titrated H_2_O_2_ concentration versus time, from solutions of different initial [H_2_O_2_] at pH = 8.7 for the system (a) PAla-Cu(II), [PAla] = 2.0 × 10^−3^ M, [Cu^2+^] = 5.0 × 10^−4^ M. *T* = 298 K. (b) PGlygly-Cu(II), [PGlygly] = 2.0 × 10^−3^ M, [Cu^2+^] = 5.0 × 10^−4^ M. *T* = 298 K.

**Figure 2:**
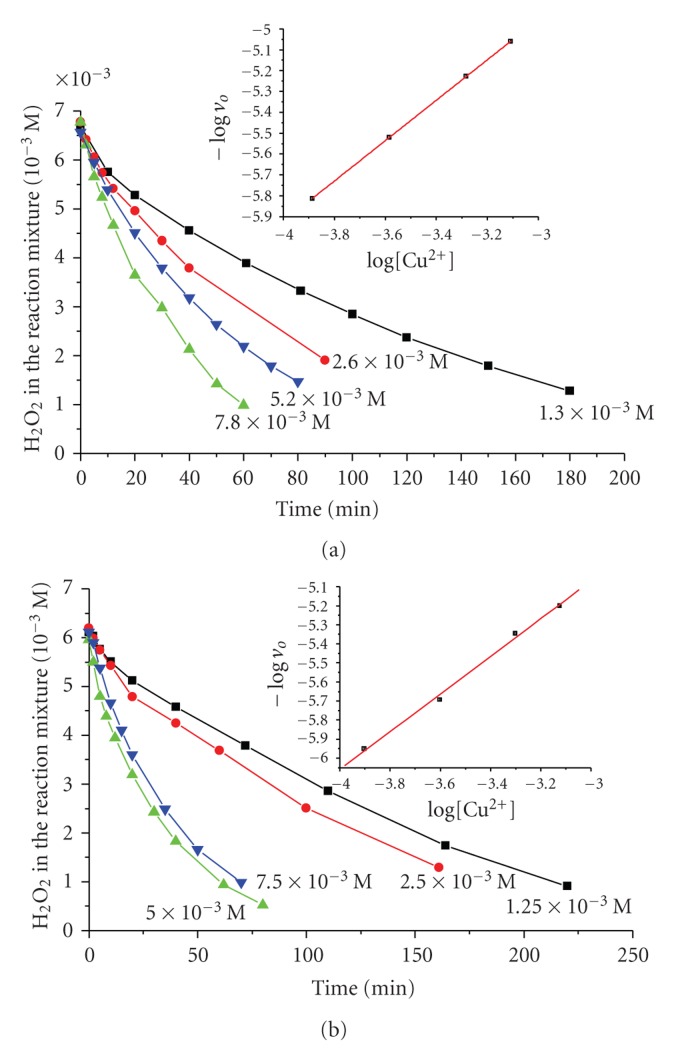
The variation of [H_2_O_2_] in the reaction mixture (% of the initial concentration) versus time for different Cu(II) concentrations, and (insert) logarithm of the initial rate (*v*_*o*_) versus logarithm of the [Cu(II)] for the system (a) PAla-Cu(II), *R* = [PAla]/[Cu(II)] = 4, initial concentration [H_2_O_2_] = 6.8 × 10^−3^ M, pH = 8.5, *T* = 298 K. (b) PGlygly-Cu(II), *R* = [PGlygly]/[Cu(II)] = 4, initial concentration [H_2_O_2_] = 6.2 × 10^−3^ M, pH = 8.9, *T* = 298 K.

The reactions are found to be of pseudo-first order with respect to H_2_O_2_ (Figure 1) for all the studied pH and [H_2_O_2_]/catalyst ratios. The order of the reactions was not found to decrease with the increase of the initial H_2_O_2_ concentration. The reactions were, also, found to be of pseudo-first order with respect to the total concentration of Cu(II) (Figure 2) for all the studied pH and [H_2_O_2_]/catalyst ratios.

**Figure 3:**
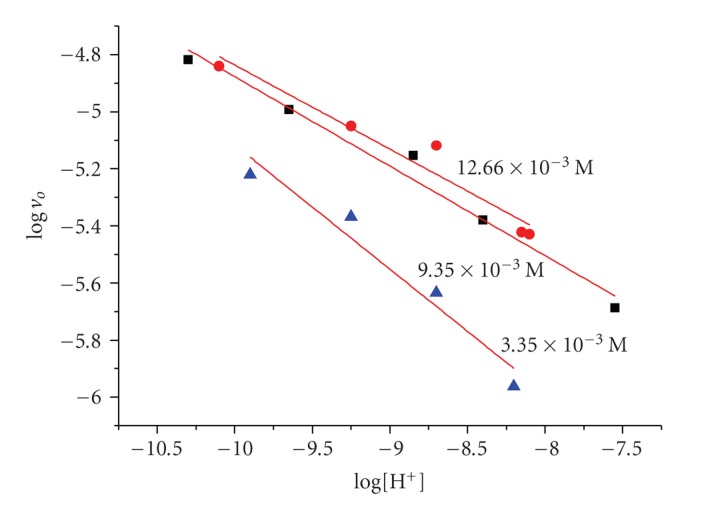
The variation of the logarithm of the initial rate (*v*_*o*_) versus logarithm of [H^+^] at different hydrogen peroxide concentrations for the system PAla-Cu(II), [PAla] = 2.0 × 10^−3^ M, [Cu^2+^] = 5.0 × 10^−4^ M. *T* = 298 K.

The dependence of the reaction rates on pH is given in Figure 3. The rates of H_2_O_2_ decomposition are pH-dependent. The observed rates {*d*[H_2_O_2_]/*d* *t*}_*t*→0_obs__ are very low below pH:8 and strongly increase in the alkaline region. The order was found to be between −0.4 and −0.8 with respect to [H^+^], depending on the [H_2_O_2_]. A possible explanation is discussed forward in the text.

#### 3.2.1. Effect of Temperature Activation Parameters

**Figure 4:**
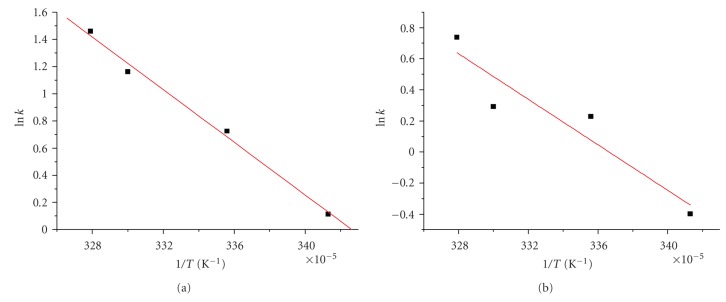
Arrhenius plots for the system (a) PAla-Cu(II), [PAla] = 2.0 × 10^−3^ M, [Cu^2+^] = 5.0 × 10^−4^ M, [H_2_O_2_] = 6.7 × 10^−3^ M, pH = 8.8 (b) PGlygly-Cu(II), [PGlygly] = 2.0 × 10^−3^ M, [Cu^2+^] = 5.0 × 10^−4^ M, [H_2_O_2_] = 6.7 × 10^−3^ M, pH = 8.8.

Using Arrhenius plots (Figure 4) and the Arrhenius equation the activation energies, *E*_*α*_, were calculated at pH 8.8 (Table 1). 

The difference in the activation energy values between the PGlygly-Cu(II) system and the PAla-Cu(II) system should reflect not only the different microenvironment of the Cu(II) ions, but also the differences of the macromolecular ligands. In these two systems, key feature is the coordination of the ligand derived from alanine to each Cu(II) through deprotonated amide nitrogens and carboxylates [35, 36] and of the ligand derived from diglycine to each Cu(II) through carboxylates [39, 46]. (Indeed, in the first coordination sphere of Cu(II) ions amidic nitrogen(s) and oxygens are involved in the PAla system, while only oxygens are involved in the case of the Pglygly.) Furthermore, the distance of the copper centers from the polymeric backbone is smaller in the PAla than in the PGlygly system. As this distance increases, not only the approach of small molecules to the metal centers is more favorable, but also any structural changes around the metals are made more easily. The different mode of complexation of the two systems is, also, reflected on the differences in the variations of the enthalpies of activation, Δ*H* ^#^, for the two systems, as Δ*H* ^#^ is a measure of the height of the energy barrier that should be overcome to reach the transition state and is related to the strengths of the intra- and intermolecular bonds which participate in the reaction leading to the transition state.

#### 3.2.2. ESR Measurements

To verify the above assumptions, the catalytic reactions that take place in solutions with pH in the range 8-9 were, also, followed with ESR spectroscopy, in order to gain some insight of the mechanism and to deduce evidence for the participating intermediates. The change of the intensity of the axial ESR signal of paramagnetic Cu(II) during the course of the reactions was monitored and the existence of any new signal, due to any newly formed paramagnetic Cu(II) species, was investigated. 

**Figure 5:**
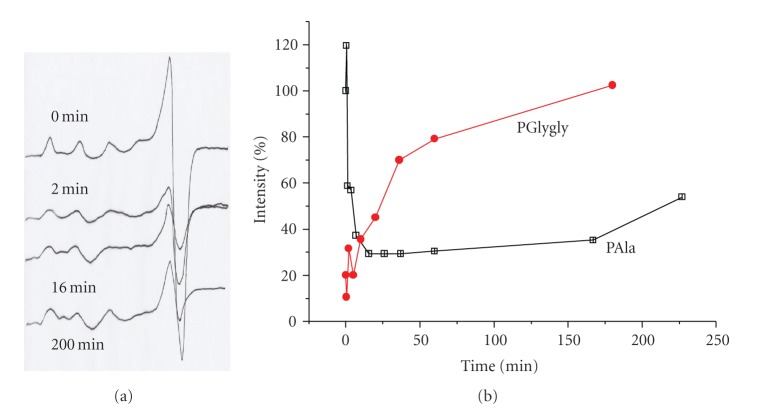
(a) EPR spectra of a frozen solution (77 K) of PAla-Cu(II) at different reaction times. [Cu(II)] = 1.0 × 10^−3^ M, *R* = [PAla]/[Cu(II)] = 4, pH = 8.7. (b) The variation of the intensity of Cu(II) signal, as (%) of the initial signal (*t* = 0 min), during the reaction time for the systems Pala-Cu(II) and PGlygly-Cu.

The two systems exhibited a completely different behavior. For the PAla-Cu(II) system we can detect three stages of the reaction (Figure 5). In the first, very short stage, a slight increase in the intensity of the signal is observed. In the second stage a rapid decrease in the intensity occurs until it reaches about 20% of the starting intensity without entire disappearance. In the final stage the signal starts building up again in a slow rate without ever reaching the original intensity. From our results it was not easy to assign any new EPR absorbing Cu(II) species, formed during the reaction. Furthermore, as any additional spectral lines in the area of *g* = 4 were not detected, any Cu(II)-Cu(II) strong antiferromagnetic coupling was precluded to be the reason for the decrease in the intensity of the signal [47, 48]. Finally, as the formation of a diamagnetic Cu(III) complex (d^8^ low-spin complex) can also be ruled out, since it requires ligands able to give strong fields as tetrapeptides or ligands able to give a four-nitrogen in plane coordination [49], and no evidence of ESR lines due to paramagnetic Cu(III) complexes could be found, the reduction of EPR-silent Cu(I) complex is strongly suggested. The small initial increase in the intensity of the signal could be explained as the result of the adoption from the negatively charged macromolecular catalyst of a more extended structure that enables the approach of the, also, negatively charged peroxo anion that comes from the dissociation of H_2_O_2_ that occurs at this pH. In this structure, the metallic centers are further apart from each other and any small spin exchange between them is diminished. 

The system PGlygly-Cu(II) shows a signal with an initial intensity of 20% of the initial intensity of the signal of the system PAla-Cu(II), for the same total Cu(II) concentration. The signal does not decrease in intensity as the reaction proceeds, but instead constantly increases. It finally reaches a plateau when the intensity equals the initial intensity of the signal of the PAla-Cu(II) system. This striking difference could not be explained only by the different nature of the catalytic sites (Cu(II) complexes), without taking into account that these sites are a part of a three-dimensional macromolecule. As the experimental conditions (pH, ionic strength, Cu(II) and polymer concentrations, temperature) of the formation of the complexes as well as the anchoring groups (amides and carboxylates) are exactly the same, the difference in the modes of complexation must be related to conformational effects. Indeed the complex formation between a polyelectrolyte and a metal ion causes many effects, mainly arising from changes in the net charge of the polymer and changes in the electrostatic interactions [2, 50, 51]. In previous works we have reported the effect of the complexation of Cu(II) ions on the hydrodynamic behavior of the PAla and PGlygly [39, 52]. In these works viscosimetric studies clearly demonstrated the formation of intramolecular Cu(II)-carboxylates complexes, which lead to the folding of the macromolecules. At higher pH values, when amide nitrogens replace carboxylates on the coordination sphere of Cu(II), only neighboring side chains are involved in complex formation, resulting in the unfolding of the macromolecules.

As a result of the differences in the complexation modes and the macromolecular ligand conformations of the two systems at the pH range under investigation, the mutual approach of the copper centers is feasible for PGlygly-Cu(II) complex but not for PAla-Cu(II). Consequently extensive Cu(II)-Cu(II) spin interactions may arise only in the PGlygly-Cu(II) system. These spin interactions explain the much smaller intensity of the initial signal in the PGlygly-Cu(II) system, as has been observed in other systems where copper-copper dipole interactions are promoted by the ligands [53, 54]. No signals were observed near *g* = 4 and *G* = (*g*_//_ − 2)/(*g*_⊥_ − 2) is greater than 4, indicating negligible exchange interactions [48, 55]. The approach of H_2_O_2_ and complexation to Cu(II) cause the unfolding of the macromolecule, as has been observed elsewhere [56], breaking the existing intra- and inter-crossings between the chains of the polymer. This results in the reduction of the spin interactions between copper centers and consequently the increase of the intensity of the paramagnetic signal. Obviously, the magnitude of the increase is bigger than the magnitude of the simultaneous decrease due to the reduction of Cu(II) to Cu(I). Furthermore, it seems that the reoxidation step {Cu(I)  to  Cu(II)} is much quicker for the PGlygly polymer, as after 3 hrs from the beginning of the reaction all the copper is in the Cu(II) state, while for the PAla polymer only 20% of the copper is in the Cu(II) state at the same time. 

We did not detect any new signals due to different from the initial Cu(II) species, for the period of a week. Thus, our experimental data cannot support the degradation process that has been reported for other systems [57].

#### 3.2.3. Kinetics and Mechanism

Kinetic studies in the conditions mentioned above showed that the initial rate of the decomposition of H_2_O_2_ is proportional to [H_2_O_2_], [CuL_2_]. For a given pH stands

(1) $$v_{o}=-d\frac{\left[{\text{H}}_{2}{\text{O}}_{2}\right]}{dt}=k_{\text{obs}}\left[{\text{CuL}}_{2}\right]\left[{\text{H}}_{2}{\text{O}}_{2}\right],$$

(*k*_obs_ depends on the pH of the solution).

The following reactions are consistent with the above-mentioned observed rate law and the thermodynamic and spectroscopic measurements

(2) $$2{\text{H}}_{2}{\text{O}}_{2}⇆2\text{HO}{\text{O}}^{-}+2{\text{H}}^{+},$$

(3) $${\left[\text{Cu}{\left(\text{L}\right)}_{2}\right]}^{-}+\text{HO}{\text{O}}^{-}⇆{\left[\text{Cu}{\left(\text{L}\right)}_{2}\left(\text{HOO}\right)\right]}^{2-},$$

(4) $${\left[{\text{Cu}}^{\text{II}}{\left(\text{L}\right)}_{2}(\text{HOO})\right]}^{2-}⇆{\left[{\text{Cu}}^{\text{I}}{\left(\text{L}\right)}_{2}({\text{HOO}}^{.})\right]}^{2-},$$

(5) $${\left[{\text{Cu}}^{\text{I}}{\left(\text{L}\right)}_{2}({\text{HOO}}^{.})\right]}^{2-}+{\text{H}{\text{O}}_{2}}^{-}\to {\left[{\text{Cu}}^{\text{II}}{\left(\text{L}\right)}_{2}\right]}^{-}+{\text{O}}_{2}+2\text{H}{\text{O}}^{-},$$

(6) $$2{\text{H}}^{+}+2\text{H}{\text{O}}^{-}⇆2{\text{H}}_{2}\text{O}.$$

It is assumed that step (4) is the rate determining step of the reaction, as the reduction of Cu(II) requires geometrical changes around the metal center. According to the Franck-Condon principle, before the electron-transfer the coordinate bonds between the cupric ion and the ligands must be stretched and the cupric complex must be rearranged to a structure which can accept one electron. This mechanism is in agreement with the suggestion for other macromolecular systems [21] and can explain the change in rate in going to more alkaline conditions, as the dependence of the redox potential of the couple HO_2_^.^ (O_2_^.−^)/H_2_O_2_ (HO_2_^−^) on pH is very well known. The redox potential drops from 1.4 to 0.18 V over the pH range 0–14 [21]. It is obvious that the oxidation of H_2_O_2_ to HO_2_^.^ or O_2_^.−^ by Cu(II) is more favorable in more alkaline conditions. This kind of mechanism does not involve any diffusible radical species in accordance to previous proposed mechanism [10, 18], but several very rapid intermediate steps between (4) and (5) may exist which include the participation of radicals. This should explain the nonlinear dependence of the initial rate of the reaction from [H^+^] as well as the formation of the brown peroxo-copper precipitate at high H_2_O_2_ concentrations, but from our data no conclusion can be drawn about the possible structures of these intermediate species.

## 4. Conclusions

The complexes of copper (II) with the functional polymers PAla and PGlygly act as catalysts in the decomposition of H_2_O_2_ at alkaline conditions. The trend in catalytic efficiency is in the order PGlygly > PAla, due to differences in the modes of complexation and the adopted conformations of the macromolecules upon complexation. The catalytic decomposition depends upon the concentration of H_2_O_2_, the concentration of the catalyst, temperature, and pH of the reacting solutions. The rate of the reaction is of pseudo-first order with respect to the concentration of H_2_O_2_ and the concentration of the catalyst at the pH range 7–10. The proposed mechanism involves reduction of Cu(II) to Cu(I), during the slow rate determining step.

## Figures and Tables

**Table 1:** Calculated energy parameters for the decomposition of H_2_O_2_ from the systems PAla-Cu(II) and PGlygly-Cu(II), respectively. *T* = 298 K, [*C* *u* ^2+^] = 5.2 × 10^−4^ M, [*P* *A* *l* *a*] = 2.0 × 10^−3^ M, [*P* *G* *l* *y* *g* *l* *y*] = 2.0 × 10^−3^ M, *R* = 4, [*H*_2_ *O*_2_] = 6.7 × 10^−3^ M, *p* *H* = 8.8.

	*E*_*α*_^#^	Δ*G* ^#^	Δ*H* ^#^	Δ*S* ^#^
	(Kcal · mol^−1^)	(Kcal · mol^−1^)	(Kcal · mol^−1^)	(cal · K^−1^ · mol^−1^)
PAla-Cu(II)	19.2 (±1.1)	13.6	18.6 (±1.1)	16.8
PGlygly-Cu(II)	14.6 (±3.6)	13.3	14.0 (±3.6)	2.1
